# Upregulated Palmitoleate and Oleate Production in *Escherichia coli* Promotes Gentamicin Resistance

**DOI:** 10.3390/molecules29112504

**Published:** 2024-05-25

**Authors:** Guozhu Ye, Lvyuan Fan, Yuhong Zheng, Xu Liao, Qiansheng Huang, Yubin Su

**Affiliations:** 1 Xiamen Key Laboratory of Indoor Air and Health, Key Laboratory of Urban Environment and Health, Institute of Urban Environment, Chinese Academy of Sciences, 1799 Jimei Road, Xiamen 361021, China; gzye@iue.ac.cn (G.Y.); xliao@iue.ac.cn (X.L.); 2 Department of Cell Biology & Institute of Biomedicine, National Engineering Research Center of Genetic Medicine, MOE Key Laboratory of Tumor Molecular Biology, Guangdong Provincial Key Laboratory of Bioengineering Medicine, College of Life Science and Technology, Jinan University, Guangzhou 510632, China; fly13542982214@163.com (L.F.); zhengyuhong2024@sibcb.ac.cn (Y.Z.)

**Keywords:** antibiotic resistance, gentamicin, metabolomics, amino acid metabolism, lipid metabolism, reactive oxygen species

## Abstract

Metabolic reprogramming mediates antibiotic efficacy. However, metabolic adaptation of microbes evolving from antibiotic sensitivity to resistance remains undefined. Therefore, untargeted metabolomics was conducted to unveil relevant metabolic reprogramming and potential intervention targets involved in gentamicin resistance. In total, 61 metabolites and 52 metabolic pathways were significantly altered in gentamicin-resistant *E. coli*. Notably, the metabolic reprogramming was characterized by decreases in most metabolites involved in carbohydrate and amino acid metabolism, and accumulation of building blocks for nucleotide synthesis in gentamicin-resistant *E. coli*. Meanwhile, fatty acid metabolism and glycerolipid metabolism were also significantly altered in gentamicin-resistant *E. coli*. Additionally, glycerol, glycerol-3-phosphate, palmitoleate, and oleate were separately defined as the potential biomarkers for identifying gentamicin resistance in *E. coli*. Moreover, palmitoleate and oleate could attenuate or even abolished killing effects of gentamicin on *E. coli*, and separately increased the minimum inhibitory concentration of gentamicin against *E. coli* by 2 and 4 times. Furthermore, palmitoleate and oleate separately decreased intracellular gentamicin contents, and abolished gentamicin-induced accumulation of reactive oxygen species, indicating involvement of gentamicin metabolism and redox homeostasis in palmitoleate/oleate-promoted gentamicin resistance in *E. coli*. This study identifies the metabolic reprogramming, potential biomarkers and intervention targets related to gentamicin resistance in bacteria.

## 1. Introduction

Antibiotics are widely used in humans, animals, agriculture and aquaculture, mainly as bactericides, and their use is still growing rapidly [1,2]. After administration, antibiotics and their metabolites are discharged into the environment, especially the aquatic environment, such as ground water, surface water, seawater, sewage sludge and sediments [1]. One of the most remarkable consequences of antibiotic use and its accumulation in the environment is antibiotic resistance, which has threatened the health of humans and ecosystems [3,4]. Notably, antibiotic resistance in microbes in humans and the environment is ubiquitous and spreads easily owing to the extensive diversity and short reproduction times of microbes [3,5,6]. Therefore, there is an urgent need to investigate the mechanism of antibiotic resistance in microbes. 

Studies reveal that metabolic reprogramming mediates antibiotic efficacy [7,8,9,10,11,12,13,14]. A metabolomic approach based on gas chromatography–mass spectrometry showed that glucose and alanine contents were decreased in kanamycin-resistant *Edwardsiella tarda* [15]. Subsequent mechanistic investigations revealed that exogenous glucose and/or alanine could enter the tricarboxylic acid cycle in the form of acetyl-CoA, and activate related metabolic enzymes in the pathway for the intermediate synthesis in *Edwardsiella tarda*, and that the activation of the tricarboxylic acid cycle by substrate stimulation increased generation of reduced nicotinamide adenine dinucleotide and proton motive force, and promoted antibiotic uptake, thus restoring the sensitivity of antibiotic-resistant bacteria [15]. Additionally, triglyceride accumulation occurred in *Mycobacterium tuberculosis* and relevant environmental bacteria under stress-induced antibiotic tolerance, such as hypoxia, low pH, iron limitation and infection [10,16]. Deletion of *tgs1*, overexpression of *citA* and ^14^C-acetate tracer experiment indicated that triglyceride synthesis directed carbon sources away from the tricarboxylic acid cycle and other growth-promoting pathways, leading to decreases in bacterial growth and antibiotic efficacy, and that disruption of the metabolic switch disabled the growth arrest responding to stress, and resensitized bacteria to antibiotic treatment during infection [10]. Moreover, the activation of glycolysis, pathways (such as the respiratory chain) that generate reactive oxygen species (ROS), or nucleotide oxidation, suppression of gluconeogenesis, and supply of some exogenous amino acids (such as glycine, serine, valine and alanine) were found to be able to improve antibiotic efficacy [8,9,11,17,18]. Above data demonstrate that reprogramming a metabolism is an effective way to improve antibiotic efficacy.

Bacteria can acquire antibiotic resistance via genetic mutation, expression and transfer of resistance gene, and/or phenotypic adaptation, which will induce metabolic disorders, and then impair relevant physiological efficiency [19]. Nonetheless, the high capability of bacteria to compensate for, attenuate or even abolish these metabolic burdens of antibiotic resistance causes stability and survival of resistant bacterial populations [20,21]. Studying how bacteria develop antibiotic resistance and discovering potential biomarkers to determine whether bacteria have developed antibiotic resistance will help us use antibiotics more effectively, and prevent and reduce the damage caused by antibiotics and their resistance. In this study, *Escherichia coli* were continuously exposed to gentamicin until they gained a gentamicin-resistant phenotype. Next, an untargeted metabolomics approach employing gas chromatography–mass spectrometry was used to uncover vital metabolic reprogramming, potential biomarkers and intervention targets associated with the evolution of *E. coli* from gentamicin-sensitive to -resistant bacteria. Furthermore, roles of some potential biomarkers in gentamicin resistance in *E. coli* were examined.

## 2. Results

### 2.1. Changes in the Metabolic Profiling of Gentamicin-Resistant E. coli

**Figure 1:**
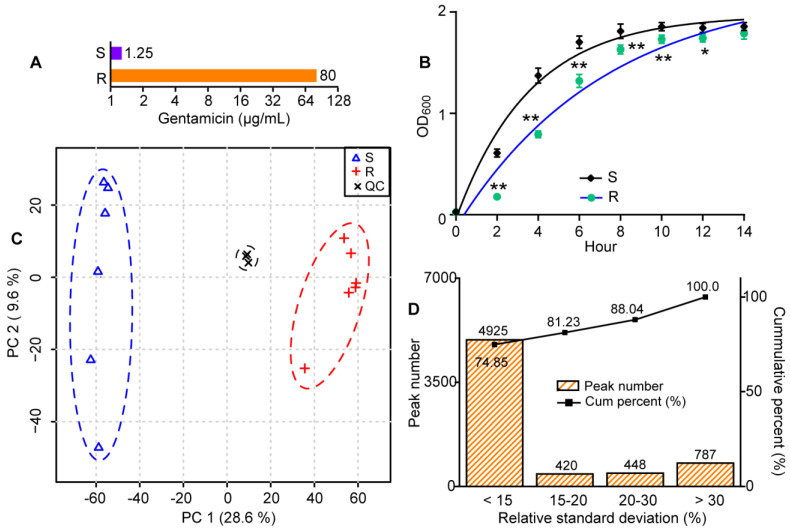
Changes in the metabolic profiles of gentamicin-resistant *E. coli*. (**A**) The MIC of gentamicin-resistant *E. coli*. (**B**) The growth curve of gentamicin-resistant *E. coli*. n = 4 per group. *, *p* < 0.05, **, *p* < 0.01, two-tailed independent samples *t*-test. (**C**) Changes in the metabolic profiles of gentamicin-resistant *E. coli*. S, gentamicin-sensitive *E. coli*; R, gentamicin-resistant *E. coli*. n = 6, 6 and 3 in the S, R and QC group, respectively. (**D**) The reproducibility and stability of detected ions in QC samples.

We found that the minimum inhibitory concentration (MIC) of gentamicin-resistant *E. coli* was 80 µg/mL, which was 64 times that of the control group (Figure 1A). The growth curve analysis showed that the growth of gentamicin-resistant *E. coli* at 2, 4, 6, 8, 10 and 12 h was significantly lower than that of the control group, and that there was no significant difference between the two groups after 14 h (Figure 1B). The data showed that gentamicin-resistant *E. coli* grew more slowly than the control group. Subsequently, gentamicin-resistant *E. coli* and its control in the stable phase were collected for untargeted metabolomic analysis to discover metabolic reprogramming and potential biomarkers related to gentamicin resistance in *E. coli*.

Principal component analysis showed that three QC samples were distributed together in the score plot (Figure 1C). In addition, among the 6580 ions detected in QC samples, there were 4925 (74.85%), 5345 (81.23%) and 5793 (88.04%) ions with relative standard deviations of their contents lower than 15, 20 and 30%, respectively (Figure 1D). These data indicated that the untargeted metabolomic approach in this study had high repeatability and stability [22,23]. Moreover, there were significant differences in the metabolic profiling between gentamicin-resistant *E. coli* and the control group, which indicated significant metabolic changes in gentamicin-resistant *E. coli* compared to the control group (Figure 1C).

### 2.2. Metabolic Reprogramming in Gentamicin-Resistant E. coli

**Figure 2:**
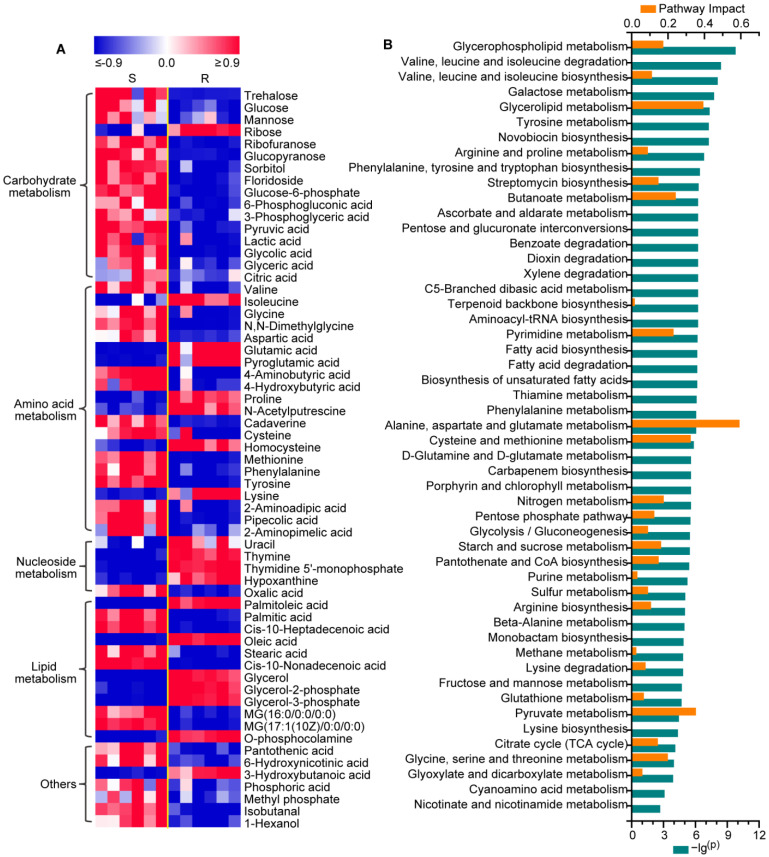
Metabolic reprogramming in gentamicin-resistant *E. coli*. (**A**) Heat map plot of metabolic reprograming in gentamicin-resistant *E. coli*. After data normalization to unit variance, the metabolite level was employed for the heat map plot. n = 6 per group. Metabolites significantly altered in gentamicin-resistant *E. coli* compared to the control (*p* < 0.05, two-tailed Mann–Whitney U test) are all shown in the plot. S, gentamicin-sensitive *E. coli*; R, gentamicin-resistant *E. coli*. (**B**) Changes in metabolic pathways. All pathways significantly altered in gentamicin-resistant *E. coli* compared to the control (*p* < 0.05) are shown.

Totally, 61 of 82 identified metabolites were found to be significantly altered in gentamicin-resistant *E. coli* compared to the control group, including 16, 21, 5, 12 and 7 metabolites involved in carbohydrate metabolism, amino acid metabolism, nucleotide metabolism, lipid metabolism and other metabolic pathways, respectively (Figure 2A and Table S1). Of the 61 differential metabolites, 45 metabolites were further confirmed by the reference standards (Table S1). Notably, levels of metabolites involved in carbohydrate metabolism were significantly decreased in gentamicin-resistant *E. coli*, including disaccharide (trehalose), monosaccharides (e.g., glucose, mannose and sorbitol), phosphorylated saccharides (e.g., glucose-6- phosphate and 6-phosphogluconate) and organic acids (e.g., pyruvate, lactate, citrate and glycerate), except ribose. In contrast, levels of metabolites involved in the synthesis of nucleic acid, including nucleotides (uracil, thymine, thymidine 5′-monophosphate and hypoxanthine) and ribose, were significantly increased in gentamicin-resistant *E. coli*. Moreover, amino acid and lipid metabolism were also significantly altered in gentamicin-resistant *E. coli*.

Subsequent pathway analysis showed that 52 metabolic pathways were significantly altered in gentamicin-resistant *E. coli*, e.g., glycerophospholipid metabolism, glycerolipid metabolism, pyrimidine metabolism, fatty acid biosynthesis, alanine, aspartate and glutamate metabolism, cysteine and methionine metabolism, pyruvate metabolism and pentose phosphate pathway (Figure 2B). Among the significantly altered metabolic pathways in gentamicin-resistant *E. coli*, the top 6 metabolic pathways with the greatest impact were as follows: alanine, aspartate and glutamate metabolism, glycerolipid metabolism, pyruvate metabolism, cysteine and methionine metabolism, butanoate metabolism and pyrimidine metabolism. Detailed metabolic reprogramming in gentamicin-resistant *E. coli* is described and discussed below.

### 2.3. Reprogramming of Carbohydrate and Nucleotide Metabolism in Gentamicin-Resistant E. coli

**Figure 3:**
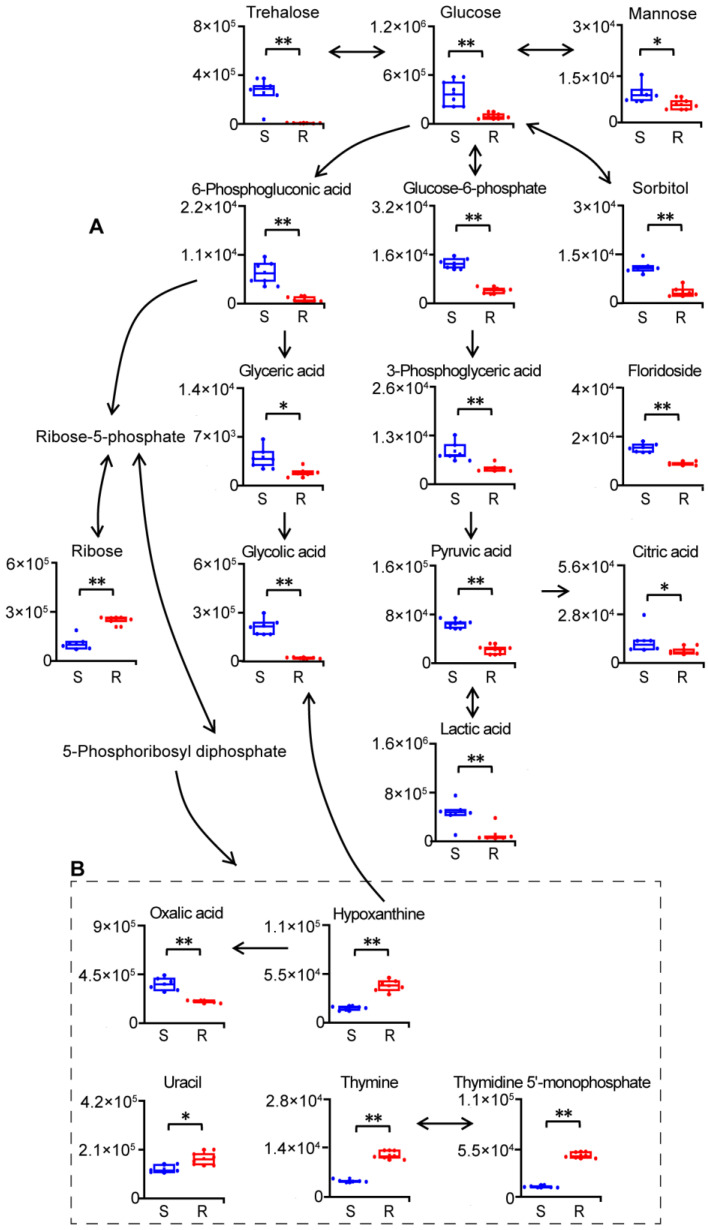
Reprogramming of carbohydrate (**A**) and nucleotide (**B**) metabolism in gentamicin-resistant *E. coli*. S, gentamicin-sensitive *E. coli*; R, gentamicin-resistant *E. coli*. n = 6 per group. *, *p* < 0.05, **, *p* < 0.01, two-tailed Mann–Whitney U test.

We found that the level of glucose, an important carbon source, and those of its interconvertible saccharides, such as trehalose, mannose and sorbitol, were significantly decreased in gentamicin-resistant *E. coli* (Figure 3A). Consistently, the level of floridoside, which is related to carbohydrate transport, assimilation and storage, was also significantly decreased in gentamicin-resistant *E. coli* (Figure 3A). These data suggested metabolic disturbances in saccharides in gentamicin-resistant *E. coli*. In addition to saccharides, levels of other metabolites involved in glycolysis, such as glucose-6-phosphate, 3-phosphoglyceric acid, pyruvic acid and lactic acid, were significantly decreased as well, which indicated the suppression of glycolysis in gentamicin-resistant *E. coli* (Figure 3A). We also observed decreased levels of glycerate, glucose-6-phosphate and 6-phosphogluconic acid, which suggested inhibition of the pentose phosphate pathway in gentamicin-resistant *E. coli* (Figure 3A). Moreover, the level of citric acid, an important intermediate in the tricarboxylic acid cycle, was significantly decreased in gentamicin-resistant *E. coli* (Figure 3A).

We also discovered that levels of metabolites involved in purine and pyrimidine nucleotide metabolism, including ribose, hypoxanthine, uracil, thymine and thymidine 5′-monophosphate, were significantly increased, while levels of the products of hypoxanthine, including oxalic acid and glycolic acid, were significantly decreased in gentamicin-resistant *E. coli* (Figure 3A,B). The accumulation of metabolites involved in the nucleotide metabolism indicated more biomaterials available for the synthesis of nucleic acid and/or energy in gentamicin-resistant *E. coli*. In addition, decreases in levels of products of hypoxanthine suggested a potential decrease in the degradation of nucleotides in gentamicin-resistant *E. coli*.

### 2.4. Reprogramming of Amino Acid Metabolism in Gentamicin-Resistant E. coli

**Figure 4:**
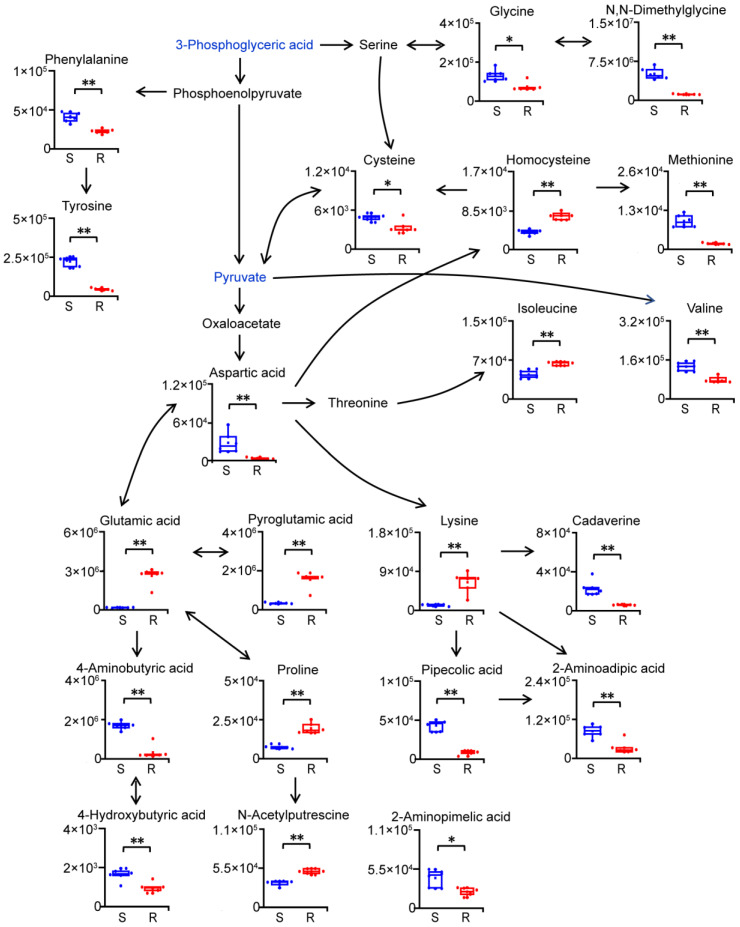
Reprogramming of amino acid metabolism in gentamicin-resistant *E. coli*. S, gentamicin-sensitive *E. coli*; R, gentamicin-resistant *E. coli*. n = 6 per group. *, *p* < 0.05, **, *p* < 0.01, two-tailed Mann–Whitney U test. Blue fonts: metabolites significantly decreased in gentamicin-resistant *E. coli*, which are shown in Figure 3.

Among significantly altered metabolic pathways in gentamicin-resistant *E. coli*, alanine, aspartate and glutamate metabolism was found to be the pathway with the largest impact. Levels of aspartic acid, 4-aminobutyric acid and 4-hydroxybutyric acid were significantly decreased, while those of glutamic acid, pyroglutamic acid, proline and N-acetylputrescine were significantly increased in gentamicin-resistant *E. coli* (Figure 4). These data suggest more glutamic acid for the synthesis of glutathione, nucleotides and proline, but less glutamic acid for the synthesis of 4-aminobutyric acid in gentamicin-resistant *E. coli*. In addition, among metabolic pathways with significant alterations in gentamicin-resistant *E. coli*, cysteine and methionine metabolism was the fourth most influential pathway. Levels of cysteine, methionine, glycine and N,N-dimethylglycine were significantly decreased, while that of homocysteine was significantly increased in gentamicin-resistant *E. coli* (Figure 4). Changes in levels of metabolites involved in cysteine and methionine metabolism, and glycine, serine and threonine metabolism indicated potential disorders of the methylation processes and/or antioxidant capability in gentamicin-resistant *E. coli*.

We also discovered significant changes in branched-chain and aromatic amino acids in gentamicin-resistant *E. coli* (Figure 4). Levels of valine, phenylalanine, and tyrosine were significantly decreased, while the isoleucine level was significantly increased in gentamicin-resistant *E. coli*. Additionally, lysine metabolism was found to be significantly altered in gentamicin-resistant *E. coli* (Figure 4). The level of lysine was significantly increased, while those of its products, including cadaverine, 2-aminoadipic acid and pipecolic acid, were significantly decreased in gentamicin-resistant *E. coli*. These data suggested a decrease in lysine degradation in gentamicin-resistant *E. coli*.

### 2.5. Reprogramming of Lipid Metabolism in Gentamicin-Resistant E. coli

**Figure 5:**
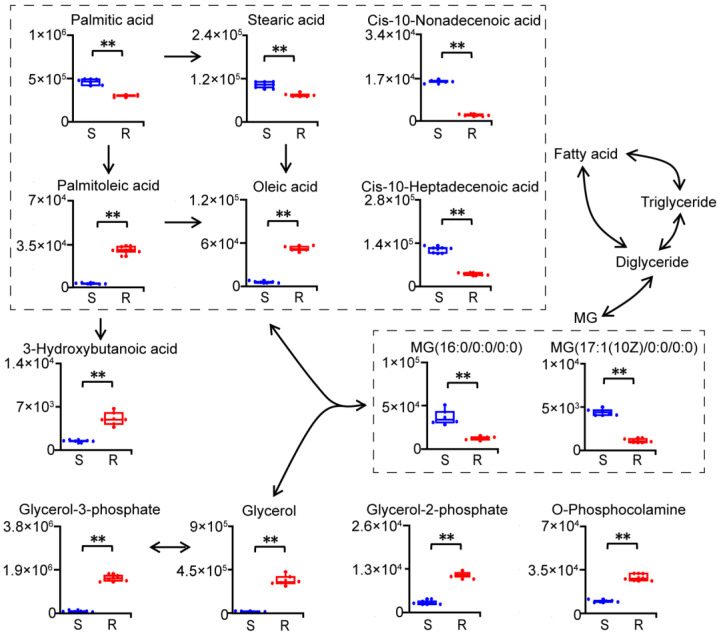
Reprogramming of lipid metabolism in gentamicin-resistant *E. coli*. S, gentamicin-sensitive *E. coli*; R, gentamicin-resistant *E. coli*; MG, monoglyceride. n = 6 per group. **, *p* < 0.01, two-tailed Mann–Whitney U test.

Lipid metabolism was significantly altered in gentamicin-resistant *E. coli* (Figure 5). Levels of palmitic acid, cis-10-heptadecenoic acid, stearic acid and cis-10-nonadecenoic acid were significantly decreased, whereas those of palmitoleic acid and oleic acid were significantly increased in gentamicin-resistant *E. coli*. Increases in palmitoleate and oleate, and decreases in palmitate and stearate, suggested potential increases in the activities of fatty acid desaturases in gentamicin-resistant *E. coli*. Meanwhile, the increased level of 3-hydroxybutanoic acid indicated potential disordered synthesis and degradation of ketone bodies, which could be synthesized from the degradation of fatty acids and/or branched-chain amino acids in gentamicin-resistant *E. coli*.

Significant changes in glycerolipid metabolism, a metabolic pathway with the second largest impact, in gentamicin-resistant *E. coli* were also observed. Levels of monoglycerides (16:0 and 17:1) were significantly decreased, while those of glycerol, glycerol-2-phosphate and glycerol-3-phosphate were significantly increased in gentamicin-resistant *E. coli*. These data indicated a disorder of glycerolipid metabolism in gentamicin-resistant *E. coli*. Moreover, the increased level of O-phosphocolamine indicated disorders of glycerophospholipid metabolism, sphingolipid metabolism and/or lipopolysaccharide modifications in gentamicin-resistant *E. coli*.

### 2.6. Potential Biomarkers Related to Gentamicin Resistance in E. coli

**Figure 6:**
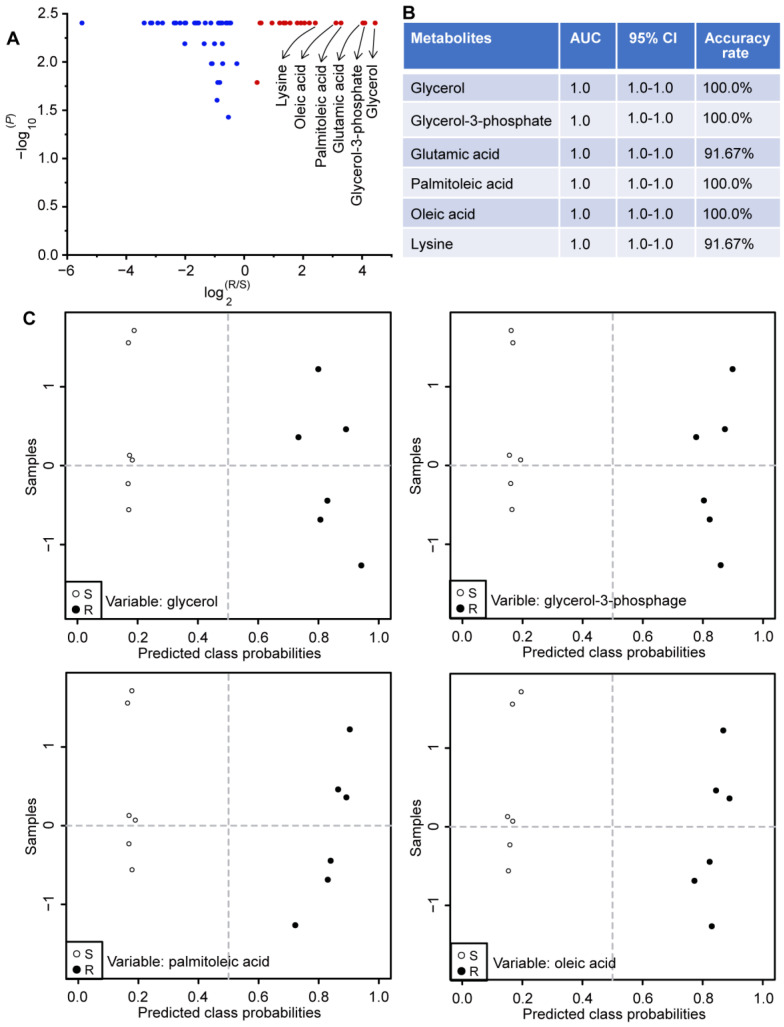
Potential biomarkers related to gentamicin resistance in *E. coli*. (**A**) Volcano plot of metabolic changes in gentamicin-resistant *E. coli*. Only the significantly altered metabolites are shown (*p* < 0.05, two-tailed Mann–Whitney U test). (**B**) The diagnostic performances of the top 6 metabolites with the largest increases in the evaluation of gentamicin resistance in *E. coli*. The values of area under the curve (AUC) and related confidence interval (CI) were calculated by performing a classical univariate receiver operating characteristic curve analysis. The accuracy rate was calculated from the sample discrimination result using the corresponding potential biomarker via the support vector machine algorithm. (**C**) The potential biomarker for evaluating gentamicin resistance in *E. coli*. Metabolites were used separately in the model of support vector machines for identification of gentamicin resistance. S, gentamicin-sensitive *E. coli*; R, gentamicin-resistant *E. coli*. n = 6 per group.

Identification of bacterial resistance would enable us to adopt more appropriate strategies and measures to improve the therapeutic efficacy of antibiotics and other drugs, and to reduce related drug use, environmental emissions and potential ecotoxicology. Accordingly, we sought to examine whether there were metabolites that could be used as indicators of gentamicin resistance in *E. coli* (Figure 6). It was clear from the volcano plot that glycerol, glycerol-3-phosphate, glutamic acid, palmitoleate, oleate and lysine were the top six metabolites with the largest increases and the lowest *p* values (Figure 6A). Therefore, a classical univariate receiver operating characteristic curve analysis was firstly used to evaluate the diagnostic performances of the above six metabolites on the assessment of gentamicin resistance in *E. coli*. It was revealed that values of the area under the curve and their corresponding confidence interval for glycerol, glycerol-3-phosphate, glutamic acid, palmitoleate, oleate and lysine were all 1.0 and [1.0, 1.0], respectively (Figure 6B). Afterwards, glycerol, glycerol-3-phosphate, glutamic acid, palmitoleate, oleate and lysine were separately used as potential biomarkers for predicting gentamicin resistance in *E. coli* using the support vector machine algorithm. The sample discrimination results demonstrate that the separate use of glycerol, glycerol-3-phosphate, palmitoleate and oleate as potential biomarkers could clearly distinguish gentamicin-resistant *E. coli* from its control, and the accuracy rate of classification was 100.0% (Figure 6B,C). However, when glutamate and lysine were separately used as potential markers for the diagnosis of gentamicin resistance in *E. coli*, one gentamicin-resistant *E. coli* sample was incorrectly identified, with the correct rate of 91.67%. Taken together, glycerol, glycerol-3-phosphate, palmitoleic acid and oleic acid were highly correlated with gentamicin resistance in *E. coli* and could be used separately as potential biomarkers for identifying gentamicin resistance in *E. coli*.

### 2.7. Palmitoleate and Oleate Promote Gentamicin Resistance in E. coli

**Figure 7:**
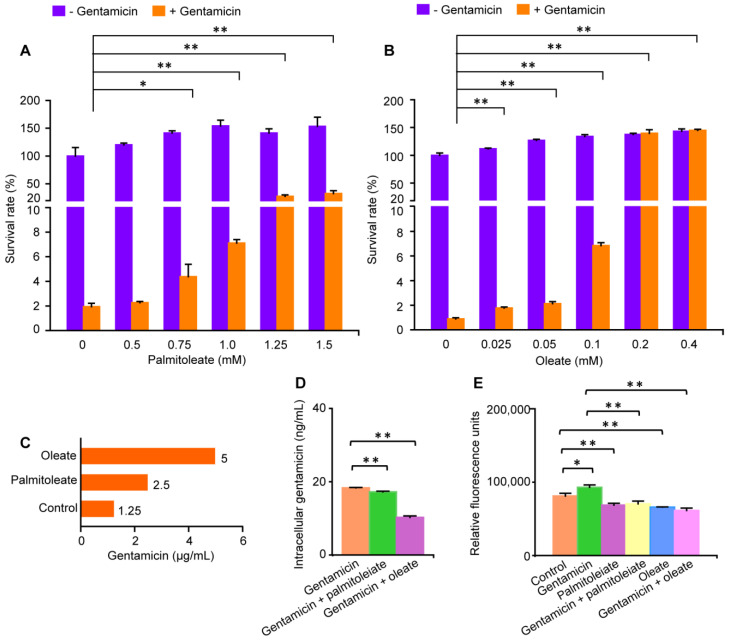
Effects of palmitoleate and oleate on gentamicin resistance in *E. coli*. Effects of palmitoleate (**A**) or oleate (**B**) on the survival rate of gentamicin-sensitive *E. coli*, the MIC of gentamicin (**C**) and intracellular contents of gentamicin (**D**) and ROS (**E**). The column represents the mean + standard deviation. n = 3 per group. *, *p* < 0.05, **, *p* < 0.01, two-tailed independent samples *t*-test.

Owing to big fold changes, high correlations with other metabolites and excellent performances in identifying gentamicin resistance in *E. coli*, potential biomarkers, glycerol, glycerol-3-phosphate, palmitoleate and oleate were examined whether they could promote gentamicin resistance in *E. coli*. We found that 0.8 μg/mL of gentamicin could effectively kill the gentamicin-sensitive *E. coli*, and that the survival rate of *E. coli* exposed to gentamicin was significantly increased by palmitoleate at concentrations from 0.75 to 1.5 mM, and could reach 27.75% and 32.63% when the concentration of palmitoleate was 1.25 and 1.5 mM, respectively (Figure 7A). In addition, the survival rate of *E. coli* exposed to gentamicin was significantly increased by oleate at concentrations from 0.025 to 0.4 mM, and could reach 139.66% and 144.97% when the concentration of oleate was 0.2 and 0.4 mM, respectively (Figure 7B). Furthermore, the MIC of gentamicin-sensitive *E. coli* cultured in the medium with 1.5 mM palmitoleate or 0.4 mM oleate was separately increased to 2 and 4 times that of the control (Figure 7C). These data indicated that both palmitoleate and oleate could promote gentamicin resistance in *E. coli*, and that only oleate could completely eliminate the killing effect of gentamicin against *E. coli*.

Since that intracellular contents of gentamicin greatly influence its killing effects and the resistance, an ELISA kit for detecting gentamicin was used. The result showed that palmitoleate and oleate could significantly reduce intracellular gentamicin contents in *E. coli* (Figure 7D). In addition, the effect of oleate was stronger than that of palmitoleate on reducing intracellular gentamicin contents in *E. coli*, which was consistent with the phenomenon that oleate had stronger effects than palmitoleate on increasing survival of *E. coli* and the MIC (Figure 7D). Given that ROS could mediate the bactericidal effect of aminoglycosides, the role of ROS in palmitoleate/oleate-promoted gentamicin resistance in *E. coli* was further explored (Figure 7E). It was revealed that gentamicin significantly increased intracellular ROS contents in *E. coli*, while palmitoleate and oleate could separately decrease intracellular ROS contents compared to the control. Moreover, combined treatments with gentamicin plus palmitoleate or oleate could effectively abolish gentamicin-induced intracellular ROS accumulation in *E. coli*. These data demonstrate the involvement of gentamicin metabolism and redox homeostasis in palmitoleate/oleate-promoted gentamicin resistance in *E. coli*. Furthermore, it was indicated that the stronger effect of oleate than that of palmitoleate on promoting gentamicin resistance in *E. coli* was involved in the gentamicin metabolism.

## 3. Discussion

We found systematic decreases in metabolites involved in carbohydrate metabolism, including the saccharide metabolism, glycolysis/gluconeogenesis, tricarboxylic acid cycle and pentose phosphate pathway, which indicated suppression of the above metabolic pathways in gentamicin-resistant *E. coli*. It was revealed that the glucose level was decreased in kanamycin-resistant *Edwardsiella tarda*, and that the supply of exogenous glucose promoted the tricarboxylic acid cycle flux and reduced nicotinamide adenine dinucleotide generation and proton driving force, thereby increasing the intake of kanamycin and restoring the sensitivity of antibiotic-resistant bacteria both in vitro and in mice with urinary tract infection [15]. In addition, it was shown that suppression of the glycolytic sugar uptake (including glucose, fructose and sucrose) by the phosphotransferase system promoted bacterial L-form growth in which bacteria were resistant to antibiotics; however, increased carbon flux through glycolysis elevated ROS generation from the respiratory chain activity, and then accelerated bacterial death, and the above effects could be overcome by either repressing the respiratory chain activity or activating the oxidative stress response genes [17]. Moreover, the activation of gluconeogenesis prevented bacterial killing by β-lactam antibiotics [17]. Consistently, increases in the gluconeogenetic flux and the pentose phosphate pathway, decreases in the biogenesis of peptidoglycan and lipopolysaccharide and disturbed fluxes of respiratory chain were observed in *Acinetobacter baumannii* ATCC 19606 treated with polymyxin [24]. Previously, we discovered that enzymes linking the phosphoenolpyruvate-pyruvate-acetyl coenzyme A pathway to the tricarboxylic acid cycle (defined as the pyruvate cycle) had pivotal roles in the increased efficacy of aminoglycoside antibiotics, and that gene depletion or inhibition of the enzymes in the pyruvate cycle suppressed the tricarboxylic acid cycle even in the case of excess carbon sources in *Edwardsiella tarda* and *E. coli* [7].

Alanine, aspartate and glutamate metabolism, the pathway with the largest impact in this study, was found to be significantly altered in gentamicin-resistant *E. coli*. Decreased glutamate dehydrogenase activity and resultant inhibition of glutamate synthesis and deamination were observed in renal tubules and mitochondria isolated from rabbits treated with gentamicin, which indicated a decreased interconversion between glutamate and 2-oxoglutarate in gentamicin-resistant *E. coli* [25]. In addition, it was shown that gentamicin treatment induced increases in malondialdehyde, 4-hydroxy-2-nonenal and protein carbonyl contents, but decreases in reduced glutathione, activities of catalase and superoxide dismutase, and the total antioxidant capacity in vivo and in vitro, which could be alleviated or even abolished by some antioxidants, such as troxerutin and resveratrol [26,27,28,29]. Therefore, increases in glutamate and pyroglutamate, and decreases in glycine and cysteine in this study suggested enhanced oxidative stress, disturbed glutathione metabolism and antioxidant capacity in gentamicin-resistant *E. coli*. Moreover, the increase in glutamate in this study also indicated more biomaterials available for the synthesis of peptidoglycan in the cell wall of gentamicin-resistant *E. coli* [30]. The glutamate racemases catalyzing the synthesis of D-glutamate from its L-isomer could be used as targets for antibiotic treatment and the development of antibiotics [31].

Aspartate, another important metabolite involved in alanine, aspartate and glutamate metabolism, was discovered to be significantly decreased in gentamicin-resistant *E. coli* in this study. A gas chromatography–mass spectrometry-based metabolomics approach combined with partial least squares discriminant analysis identified aspartate as the most important feature for the identification of neomycin sulfate resistance in *Aeromonas hydrophila* [32]. Subsequent functional verification experiments showed that supply of exogenous aspartate enhanced not only the sensitivity of *Aeromonas hydrophila* and *Carassius auratus* to neomycin sulfate, but also the survival rate of fish (*Carassius auratus*) infected with *Aeromonas hydrophila* [32]. Moreover, proline, another metabolite that can be interconverted with glutamate, was found to be significantly increased in gentamicin-resistant *E. coli* in this study. The increases in proline and its precursor glutamate indicated enhanced osmoprotection to gentamicin-resistant *E. coli* via roles of the ProJ-ProA-ProH enzyme [33].

Cysteine and methionine metabolism, glycine, serine and threonine metabolism, branched-chain and aromatic amino acid metabolism were also significantly altered in gentamicin-resistant *E. coli* in this study. Functional investigations of nine amino acids (including glycine, serine, threonine, methionine, leucine, isoleucine, valine, phenylalanine and tyrosine) revealed that each of the tested amino acids had the potential to lower the viability of *Edwardsiella piscicida* EIB202 by kanamycin, but the efficacy showed differences [8]. Specifically, the effect of kanamycin was increased by 273 and 166 times by serine and glycine, approximately 5 times by phenylalanine and threonine, and about 2 times by other amino acids. The synergistic effects of exogenous glycine, threonine, serine and glucose on proton motive force and intracellular kanamycin showed that these exogenous metabolites promoted proton motive force generation and activities of pyruvate dehydrogenase, alpha-ketoglutarate dehydrogenase and succinate dehydrogenase, but decreased the survival of *Edwardsiella piscicida*, and the potentiation of these metabolites was blocked by the inhibitor of pyruvate dehydrogenase, succinate dehydrogenase, oxidases of the respiratory chain and proton motive force, respectively [8]. Moreover, exogenous cysteine was found to improve the killing of multidrug-resistant *E. coli* ATCC 25922 and *E. coli* B2 by ciprofloxacin via accelerating the tricarboxylic acid cycle, bacterial respiration and ROS generation [34].

In this study, we found that palmitoleate and oleate could alleviate and completely eliminate the antimicrobial effects of gentamicin on *E. coli* and that palmitoleate and oleate could increase the MIC of gentamicin against *E. coli*. In addition, the ratio of oleate to stearate and that of palmitoleate to palmitate were increased in gentamicin-resistant *E. coli*. Stimulated Raman scattering microscopy and lipidomics discovered increased signals of unsaturated lipids in intracellular lipid droplets, which was mainly ascribed to the high ratio of unsaturated to saturated fatty acids, especially that of oleate to stearate, within metastatic M381 melanoma cells with low metabolic activity and BRAFi resistance [35]. Subsequent functional experiments demonstrated that suppressing stearoyl-CoA desaturase-1 increased cellular lipid saturation, and consequently induced intracellular phase-separated solid membrane domains and resultant apoptosis, and that the effects of mono-unsaturation inhibition on cellular phase separation and apoptosis could be rescued via oleate treatment [35]. Moreover, it was revealed that exogenous palmitoleate and oleate mediated lipid composition and distribution in the cell membrane, enhanced lipid unsaturation and membrane fluidity, and maintained cellular morphology, surface roughness and integrity to improve resistance to salt stress in *Zygosaccharomyces rouxii* [36]. Above data suggest that palmitoleate/oleate reduced intracellular contents of gentamicin and ROS by altering the structure and function of the cell membrane, thus promoting gentamicin resistance in *E. coli* in this study. In addition, we found that palmitoleate and oleate had high sensitivity and specificity for identifying gentamicin-resistant bacteria, showing potential application prospects in the identification of aminoglycoside antibiotic resistance in bacteria. However, related applications still need to be validated by more samples, especially for the aminoglycoside-resistant bacteria developed under other conditions.

Glycerolipid metabolism, the second most influential metabolic pathway in this study, was found to be significantly altered in gentamicin-resistant *E. coli*. It was shown that diverse growth-limiting stresses, such as hypoxia, low pH or Fe, activated *tgs1*-mediated triglyceride synthesis and accumulation by directing acetyl-CoA away from the tricarboxylic acid cycle, which led to decreases in the growth and antibiotic efficacy in *Mycobacterium tuberculosis*, and that both the overexpression of *citA* and deletion of *tgs1*, which disrupted the metabolic switch, contributed to the bacterial growth responding to stress and caused antibiotic sensitivity during infection [10]. In addition, most lipids, including medium-fatty acids, branched fatty acids, monohydroxy fatty acids and lysolipids, were reduced, while monoglycerides were increased in *E. coli* under separate antibiotic treatment with ampicillin, kanamycin or norfloxacin [11]. Above data indicate potential roles of glycerolipid metabolism in gentamicin resistance in *E. coli* in this study.

## 4. Materials and Methods

### 4.1. Materials

The ultrapure water was prepared using a Milli-Q water system (Millipore Co., Boston, MA, USA). Methanol (High performance liquid chromatography grade), methoxyamine hydrochloride (98%), pyridine (99.8%) and N-methyl-N-(trimethylsilyl)-trifluoro-acetamide (≥98.5%) were purchased from Sigma-Aldrich (Shanghai, China). Palmitoleate and sodium oleate were purchased from Macklin (Shanghai, China). Gentamicin sulphate was obtained from Shanghai Sangon Biotech (Shanghai, China).

### 4.2. Bacterial Strain and Growth Condition

The bacterial strain used in this study was *E. coli* K12 BW25113, which was purchased from the Guangdong Microbial Culture Collection Center (Guangzhou, China). Gentamicin-resistant *E. coli* K12 BW25113 strain was selected from sequential propagation of *E. coli* K12 BW25113 in Luria–Bertani broth with 0.625 μg/mL (1/2 MIC) of gentamicin sulphate. Meanwhile, as the control group, gentamicin-sensitive *E. coli* K12 BW25113 was obtained by the same propagation without the antibiotic. *E. coli* was grown in 50 mL Luria–Bertani broth at 37 °C for 12 h in 250 mL flasks. Bacterial cells were collected via centrifugation at 8000 rpm at 4 °C for 5 min.

### 4.3. Growth Curve Measurement

A single clone of *E. coli* was cultured in Luria–Bertani medium without gentamicin for 12 h to reach growth saturation. Subsequently, cells were inoculated to 50 mL Luria–Bertani broth in 250 mL flasks at a ratio of 1:100 (*v*/*v*) at 37 °C in a shaker at 200 rpm. Samples were collected and their absorbance were measured every 2 h during the 14 h incubation period. The OD_600_ values were recorded, and the growth curve of the strains were drawn. There were four biological replicates.

### 4.4. Determination of MIC

The MIC value was determined via microdilution [37]. Briefly, gentamicin was gradually diluted (two-fold dilution increments) using Luria–Bertani broth in a 96-well plate, and the concentration ranged from 0.625 to 160 μg/mL. Then, 10 μL of diluted bacterial solution was added to each well of the 96-well plate (except negative control wells), so that each well contained 5 × 10^4^ colony-forming bacteria. The bacterial cell was incubated at 37 °C for 16 h, and the lowest concentration of gentamicin that could completely inhibit the growth of bacteria was observed and recorded as the MIC. The experiment was performed over three biological replicates.

### 4.5. Screening of Gentamicin-Resistant E. coli

A single bacterial colony was picked from the *E. coli* K12 BW25113 plate and inoculated into a 5 mL Luria–Bertani tube. The cells were cultured at 200 rpm at 37 °C for 12 h to reach the saturation state. The bacterial liquid was transferred to a 1 mL Luria–Bertani liquid medium with or without 1/2 MIC of gentamicin (0.625 μg/mL) at 1:100, and then incubated at 37 °C for every 12 h per generation. A single clone was obtained from every 5 generations, and the MIC value was determined. The *E. coli* was continuously exposed to gentamicin, with the concentration starting from 0.5 times MIC and increasing by two times until it obtained gentamicin resistance with 64 times MIC compared to the original strain. After that, gentamicin-resistant *E. coli* was stored at −80 °C for subsequent experiments.

### 4.6. Sample Collection for the Metabolomic Approach

Bacterial sample collection for the growth curve analysis and metabolomics approach was carried out according to the work [38]. Single colonies of gentamicin-resistant and -sensitive *E. coli* were picked from the solid Luria–Bertani plate, and inoculated into 5 mL liquid medium for 12 h. Subsequently, cells were transferred to 50 mL liquid medium at a ratio of 1:100 (*v*/*v*) and cultivated until the OD_600_ value was 1.0. A total of 10 mL cells with OD_600_ of 1.0 were washed three times with sterile saline, centrifuged at 8000 rpm for 5 min and then stored at −80 °C for subsequent experiments.

### 4.7. Sample Preparation for the Metabolomic Approach

One milliliter of 80% methanol (*v*/*v*) pre-cooled at −20 °C was added to the bacterial sample, which was then vortexed for 2 min. Sonication (200 W, 2 s pulse and 3 s pause on ice, 5 min) was used to lyse the cells. Following the centrifugation at 4 °C for 15 min at 13,000 rpm, 700 µL of the supernatant was collected for the vacuum concentration and drying in a SpeedVac concentrator (Thermo Scientific, Waltham, MA, USA). Afterwards, 50 µL of methoxyamine hydrochloride solution (20 mg/mL in pyridine) was added to the dried sample. After 30 s vortex, the oximation reaction was carried out for 1.5 h in a 37 °C water bath. Subsequently, 50 µL N-methyl-N-(trimethylsilyl)-trifluoro-acetamide was added to the sample for the silylation reaction in a 37 °C water bath for 1 h. Finally, the derivatized sample was centrifuged at 4 °C at 13,000 rpm for 15 min, and the supernatant was taken out for the instrumental analysis.

To evaluate the stability and reproducibility of the untargeted metabolomic approach in this study, the remaining supernatant of each sample extract was taken out, mixed thoroughly and then divided into 700 µL aliquots as the quality control (QC) samples. One QC sample was inserted after every 4 analytical samples. The QC sample was processed in the same way as other analytical samples in the process of vacuum drying, derivatization, instrument analysis and data processing.

### 4.8. Instrumental Analysis for the Metabolomic Approach

A gas chromatography–mass spectrometry (GCMS-QP 2010 plus, Shimadzu, Kyoto, Japan)-based untargeted metabolomics approach was employed to acquire the metabolic profiling of *E. coli*. The instrumental parameters were set according to those used in our previous works [39,40]. The injection volume, inlet temperature, split ratio of the carrier gas (high-purity helium) and constant linear velocity were set to 1 µL, 300 °C, 10:1 and 40.0 cm/s, respectively. A DB-5 MS capillary column (30 m × 250 μm × 0.25 μm, J&W Scientifc Inc., Folsom, CA, USA) was used for metabolite separation. The oven temperature was initially set to 70 °C for 3 min, and then elevated to 300 °C at a rate of 5 °C per min, which was maintained for another 10 min. Temperatures of the interface and ion source were separately set to 280 and 230 °C. The ionization mode of metabolites was electron impact, and the voltage was 70 eV. The mass signals from 33 to 600 *m*/*z* were acquired in full scan mode. The solvent delay time was 5.3 min, and the event time was 0.2 s. The detector voltage was set to the tuning voltage. The detection of a light diesel sample was conducted to obtain the retention time of n-alkanes using the same parameters of the instrumental analysis as those of analytical samples. Thereafter, retention indices of metabolites could be obtained.

### 4.9. Data Preprocessing for the Metabolomic Approach

Following the export by GCMS solution 4.2 (Shimadzu, Japan), the raw mass data in NetCDF format were used for the peak processing employing the XCMS method [41]. The full width at half maximum and the ratio of signal to noise were 4 s and 5, respectively, during the peak processing via XCMS. Feature ions of metabolites were obtained after the peak deconvolution using ChromaTOF 4.43 (LECO Cor., St. Joseph, MI, USA). The identification of metabolites was mainly based on retrieval results of commercial mass spectra libraries, followed by the manual comparison and verification by available reference standards according to the retention time, retention index and mass spectra. After normalization to the total ion current, the ion peak area was multiplied by 1 × 10^8^, and the data were applied for the following statistical analysis.

### 4.10. Antibiotic Bactericidal Assay

The bactericidal assay was carried out as previously described [42,43]. In brief, a single bacterial colony was grown in 50 mL Luria–Bertani broth in 250 mL flasks for 12 h at 37 °C. After centrifugation at 8000 rpm for 5 min, samples were washed three times with 0.85% sterile saline and re-suspended in M9 minimal media supplemented with 10 mM acetate, 1 mM MgSO_4_ and 100 μM CaCl_2_, and the OD_600_ value was adjusted to 0.2. Cells were treated with palmitoleate or oleate with or without 0.8 μg/mL of gentamicin, and incubated at 37 °C for 6 h. After that, 100 μL aliquots of samples were periodically removed, serially diluted to 10 μL aliquots and plated onto Luria–Bertani agar plates. The plates were cultured at 37 °C for 12 h. Only those dilutions yielding 20–200 colonies were enumerated to calculate colony-forming units. The survival rate was determined by dividing the colony-forming units of the treated sample by those of the control sample.

To examine the effect of palmitoleate/oleate on the MIC of gentamicin against *E. coli*, the bacteria were cultured in Luria–Bertani medium with 1.5 mM palmitoleate or 0.4 mM oleate at 37 °C. Overnight bacterial cultures were diluted at 1:100 in fresh Luria–Bertani medium with 1.5 mM palmitoleate or 0.4 mM oleate, and cultured at 37 °C to an OD_600_ of 0.6. Subsequently, bacteria were cultured in the 96-well plate at a density of 10^5^ colony-forming units/well, and then antibiotics were separately added from low to high in a 2-fold concentration gradient. After incubation at 37 °C for 12 h, the lowest concentration of antibiotics that induced no visible bacterial growth was recorded as the MIC. Three biological replicates were performed.

### 4.11. Determination of Intracellular Gentamicin

Overnight bacteria were collected and adjusted to the concentration with an OD_600_ value of 1.0, and separately treated with gentamicin (0.8 μg/mL), gentamicin (0.8 μg/mL) plus palmitoleate (1.5 mM) and gentamicin (0.8 μg/mL) plus sodium oleate (0.4 mM) for 6 h at 37 °C and 220 rpm. After the treatment, cells were harvested and washed three times with phosphate buffered saline, and lysed with sonication (200 W total power with 20% output, 2 s pulse and 3 s pause) over ice for 20 min. Following centrifugation at 12,000× *g* for 10 min at 4 °C, cells were collected for detecting intracellular gentamicin contents according to the ELISA kit (CUSABIO, Wuhan, Hubei, China).

### 4.12. Determination of ROS

2′,7′-dichlorodihydrofluorescein diacetate (DCFH-DA, Sigma, St. Louis, MO, USA) was used to measure ROS contents at an excitation wavelength of 485 nm and emission wavelength of 515 nm by employing a multifunctional enzyme-labeling instrument (Biotek, Synergy HT, Winooski, VT, USA). Overnight bacterial cultures were collected and centrifugally washed three times with 0.85% NaCl at 8000 rpm for 3 min. After the OD_600_ value was adjusted to 0.6 with the M9 minimal medium, cells were separately incubated with gentamicin (0.8 μg/mL), palmitoleic acid (1.5 mM), gentamicin (0.8 μg/mL) plus palmitoleic acid (1.5 mM), sodium oleate (0.4 mM) and gentamicin (0.8 μg/mL) plus sodium oleate (0.4 mM) at 37 °C, 220 rpm for 6 h. Afterwards, 194 µL of cells and 4 µL of 2′,7′-dichlorodihydrofluorescein diacetate (final concentration, 20 mM) were added to a 96-well plate, and incubated at 37 °C for 1 h in the dark to detect intracellular ROS contents.

### 4.13. Statistical Analysis

Two-tailed independent sample *t*-test was performed via PASW Statistics 18 (SPSS Inc., Chicago, IL, USA). Two-tailed Mann–Whitney U test and heat map plot were conducted using MultiExperiment Viewer 4.9.0 [44]. Principal component analysis, metabolic pathway analysis, classical univariate receiver operating characteristic curve analysis and support vector machine algorithm for sample classification were conducted via MetaboAnalyst 5.0 [45]. The level of statistical significance was 0.05.

## 5. Conclusions

In summary, we provided a comprehensive metabolic profile and potential biomarkers related to the evolution of *E. coli* from gentamicin-sensitive to -resistant bacteria in this study. Furthermore, palmitoleate/oleate was discovered to be able to alleviate and completely eliminate the antimicrobial effects of gentamicin on *E. coli*, respectively, and to increase gentamicin resistance in *E. coli*, which was involved in decreases in intracellular gentamicin and ROS contents. This study provides novel perspectives on the metabolic adaption of *E. coli*. evolving from gentamicin-sensitive to -resistant bacteria, potential biomarkers and relevant intervention targets, which is conductive to using antibiotics more effectively and reducing the health hazards of antibiotics and their resistance to humans and ecosystems. In addition, this study shows the applicability and power of metabolomics in discovering key metabolic reprogramming and potential intervention targets in living systems.

## Data Availability

The data presented in this study are available on request from the corresponding author.

## Supplementary Materials

The following supporting information can be downloaded at: https://www.mdpi.com/article/10.3390/molecules29112504/s1, Table S1: Changes in differential metabolites in gentamicin-resistant *Escherichia coli* compared to the control group.
