# Supplementary material for: Upregulated Palmitoleate and Oleate Production in Escherichia coli Promotes Gentamicin Resistance
Source: Molecules. 2024 May 25;29(11):2504. doi: 10.3390/molecules29112504 (PMC11173871; doi:10.3390/molecules29112504)
Supplement: Supplementary file 1 [file molecules-29-02504-s001.zip › molecules-3018893-supplementary.pdf]

Table S1. Changes in differential metabolites in gentamicin-resistant *Escherichia coli* compared to the control group.

**Metabolites**	**Related pathways**	**R/S**	***P***
Carbohydrate metabolism			
Trehalose[a]	Starch and sucrose metabolism	**0.02**	**0.004**
Glucose[a]	Glycolysis, gluconeogenesis, pentose phosphate pathway	**0.25**	**0.004**
Mannose[a]	Galactose metabolism	**0.60**	**0.010**
Ribose[a]	Pentose phosphate pathway	**2.31**	**0.004**
Ribofuranose	Carbohydrate metabolism	**0.40**	**0.004**
Glucopyranose	Carbohydrate metabolism	**0.11**	**0.004**
Sorbitol[a]	Fructose and mannose metabolism	**0.31**	**0.004**
Floridoside	Carbohydrate storage, transport, and assimilation	**0.57**	**0.004**
Glucose-6-phosphate[a]	Glycolysis	**0.32**	**0.004**
6-Phosphogluconic acid	Pentose phosphate pathway	**0.13**	**0.004**
3-Phosphoglyceric acid[a]	Glycolysis	**0.50**	**0.006**
Pyruvic acid[a]	Glycolysis	**0.34**	**0.004**
Lactic acid[a]	Glycolysis	**0.25**	**0.006**
Glycolic acid[a]	Glyoxylate and dicarboxylate metabolism	**0.10**	**0.004**
Glyceric acid[a]	Pentose phosphate pathway, glyoxylate and dicarboxylate metabolism	**0.47**	**0.010**
Citric acid[a]	Tricarboxylic acid cycle	**0.53**	**0.025**
Amino acid metabolism			
Valine[a]	Valine, leucine and isoleucine metabolism	**0.60**	**0.004**
Isoleucine[a]	Valine, leucine and isoleucine metabolism	**1.45**	**0.004**
Glycine[a]	Glycine, serine and threonine metabolism	**0.56**	**0.016**

**Metabolites**	**Related pathways**	**R/S**	***P***
N,N-Dimethylglycine	Glycine, serine and threonine metabolism	**0.22**	**0.004**
Aspartic acid[a]	Alanine, aspartate and glutamate metabolism	**0.11**	**0.004**
Glutamic acid[a]	Alanine, aspartate and glutamate metabolism	**16.32**	**0.004**
Pyroglutamic acid[a]	Alanine, aspartate and glutamate metabolism	**4.64**	**0.004**
4-Aminobutyric acid[a]	Alanine, aspartate and glutamate metabolism	**0.19**	**0.004**
4-Hydroxybutyric acid	A precursor and a metabolite of 4-aminobutyricacid	**0.60**	**0.006**
Proline[a]	Arginine and proline metabolism	**2.62**	**0.004**
N-Acetylputrescine	Arginine and proline metabolism	**1.49**	**0.004**
Cysteine[a]	Cysteine and methionine metabolism	**0.69**	**0.037**
Homocysteine[a]	Cysteine and methionine metabolism	**1.93**	**0.004**
Methionine[a]	Cysteine and methionine metabolism	**0.20**	**0.004**
Phenylalanine[a]	Phenylalanine metabolism	**0.56**	**0.004**
Tyrosine[a]	Tyrosine metabolism	**0.20**	**0.004**
Lysine[a]	Lysine metabolism	**5.32**	**0.004**
Cadaverine[a]	Lysine degradation	**0.25**	**0.004**
2-Aminoadipic acid[a]	Lysine degradation	**0.39**	**0.006**
Pipecolic acid[a]	Lysine degradation	**0.20**	**0.004**
2-Aminopimelic acid	Amino acid metabolism	**0.54**	**0.016**
Nucleotide metabolism			
Uracil[a]	Pyrimidine metabolism	**1.35**	**0.016**
Thymine[a]	Pyrimidine metabolism	**2.64**	**0.004**
Thymidine 5'-monophosphate	Pyrimidine metabolism	**4.13**	**0.004**
Hypoxanthine[a]	Purine metabolism	**2.54**	**0.004**

**Metabolites**	**Related pathways**	**R/S**	***P***
Oxalic acid	Purine degradation	**0.55**	**0.004**
Lipid metabolism			
Palmitoleic acid[a]	Fatty acid metabolism	**9.75**	**0.004**
Palmitic acid[a]	Fatty acid metabolism	**0.65**	**0.004**
Cis-10-Heptadecenoic acid	Fatty acid metabolism	**0.33**	**0.004**
Oleic acid[a]	Fatty acid metabolism	**8.66**	**0.004**
Stearic acid[a]	Fatty acid metabolism	**0.73**	**0.004**
Cis-10-Nonadecenoic acid	Fatty acid metabolism	**0.15**	**0.004**
Glycerol[a]	Glycerolipid metabolism	**21.77**	**0.004**
Glycerol-2-phosphate[a]	Glycerolipid metabolism	**3.81**	**0.004**
Glycerol-3-phosphate[a]	Glycerolipid metabolism	**17.07**	**0.004**
MG(16:0/0:0/0:0)[a]	Glycerolipid metabolism	**0.34**	**0.004**
MG(17:1(10Z)/0:0/0:0)	Glycerolipid metabolism	**0.25**	**0.004**
O-Phosphocolamine[a]	Glycerophospholipid metabolism	**2.94**	**0.004**
Others			
Pantothenic acid[a]	Pantothenateand CoA biosynthesis	**0.55**	**0.004**
6-Hydroxynicotinic acid	Nicotinate and nicotinamide metabolism	**0.71**	**0.004**
3-Hydroxybutanoic acid[a]	Synthesis and degradation of ketone bodies	**3.48**	**0.004**
Phosphoric acid[a]	Phosphonate and phosphinate metabolism	**0.84**	**0.010**
Methyl phosphate[a]	Phosphonate and phosphinate metabolism	**0.46**	**0.010**
Isobutanal	———	**0.46**	**0.004**
1-Hexanol	———	**0.67**	**0.004**

[a] Metabolites labeled with a were identified based on the search results of commercial mass spectra libraries, and manually verified, finally verified by available reference standards according to the retention time, retention index, and mass spectra; other metabolites were identified according to the search results of commercial mass spectra

libraries, and then manually confirmed. Differential metabolites were discovered by the two-tailed Mann-Whitney U test ($P < 0.05$). S, gentamicin-sensitive *E. coli*; R, gentamicin-resistant *E. coli*. Red/blue bold fonts: metabolites significantly increased/decreased in gentamicin-resistant *Escherichia coli* compared to the control group ($P < 0.05$). n = 6 per group.
